# Fabrication of ZnWO_4_/Carbon Black Nanocomposites Modified Glassy Carbon Electrode for Enhanced Electrochemical Determination of Ciprofloxacin in Environmental Water Samples

**DOI:** 10.3390/ma16020741

**Published:** 2023-01-12

**Authors:** Kiruthika Mariappan, Saranvignesh Alagarsamy, Shen-Ming Chen, Subramanian Sakthinathan

**Affiliations:** 1 Department of Chemical Engineering and Biotechnology, National Taipei University of Technology, No. 1, Section 3, Chung−Hsiao East Road, Taipei 106, Taiwan; 2 Department of Materials and Mineral Resources Engineering, National Taipei University of Technology, No. 1, Section 3, Zhong-Xiao East Road, Taipei 106, Taiwan

**Keywords:** zinc tungsten oxide, carbon black, hydrothermal method, environmental pollutant, ciprofloxacin, electrochemical sensor

## Abstract

The major problem facing humanity in the world right now is the sustainable provision of water and electricity. Therefore, it is essential to advance methods for the long-term elimination or removal of organic contaminants in the biosphere. Ciprofloxacin (CIP) is one of the most harmful pollutants affecting human health through improper industrial usage. In this study, a zinc tungsten oxide (ZnWO_4_) nanomaterial was prepared with a simple hydrothermal synthesis. The ZnWO_4_/Carbon black nanocomposites were fabricated for the determination of CIP. The nanocomposites were characterized by field emission scanning electron microscopy, energy dispersion X-ray spectroscopy, X-ray diffraction, X-ray photoelectron spectroscopy, and electrochemical impedance spectroscopy. Electrochemical studies were done using cyclic voltammetry and differential pulse voltammetry methods. Based on the electrode preparation, the electrochemical detection of CIP was carried out, producing exceptional electrocatalytic performance with a limit of detection of 0.02 μM and an excellent sensitivity of (1.71 μA μM^−1^ cm^−2^). In addition, the modified electrode displayed great selectivity and acceptable recoveries in an environmental water sample analysis for CIP detection of 97.6% to 99.2%. The technique demonstrated high sensitivity, selectivity, outstanding consistency, and promise for use in ciprofloxacin detection. Ciprofloxacin was discovered using this brand-new voltammetry technique in a water sample analysis.

## 1. Introduction

The second-generation fluoroquinolone ciprofloxacin (CIP), also known as 1-cyclopropyl-6-fluoro-1,4-dihydro-4-oxo-7-(1-piperazinyl)-3-quinoline carboxylic acid is used to treat various bacterial infections [1]. CIP is one of the most often prescribed fluoroquinolones and is widely used in people and cattle for its broad-spectrum antibacterial action. Unlike other antibiotics, CIP can be induced either parenterally or orally [2]. This medication is used to treat a variety of infectious disorders, including urinary tract, respiratory tract, and gastrointestinal tract infections, as well as skin and soft tissue infections. Furthermore, ingesting foods of animal origin in humans might result in allergic or carcinogenic responses owing to antibiotic residues [3]. However, the most severe of all the negative effects of antibiotic residues in the environment is the rise of microbial drug resistance. Additionally, it is used to treat those who have contracted anthrax following inhalation exposure [4,5]. Agranulocytosis, thrombocytopenia, myelosuppression, toxic epidermal necrolysis, swelling or tearing of a tendon, toxic epidermal necrolysis, myelosuppression, and myasthenia gravis are some of the adverse effects of the medication [6,7].

CIP overdose causes severe hepatotoxicity, and it is crucial to measure the amount of ciprofloxacin in human plasma, serum, and urine to track drug buildup in patients with hepatic dysfunction or to identify CIP poisoning in victims of severe overdose. Drug quality control also heavily relies on CIP analysis [8]. Therefore, a simple, quick, accurate approach for the measurement of CIP in pharmaceutical formulations and bodily fluids must be developed. Numerous techniques, including voltammetry, high-performance liquid chromatography, spectrophotometry, and fluorimetry, have been used for the analysis of CIP. It has a high absorption and distribution rate in fluids and tissues [9,10]. The capacity of the bacteria to grow despite the presence of antibiotics is how this phenomenon is defined. Antibiotics are not the only medication that leave traces in the environment [11]. Due to the availability of numerous medicines, including substituted acetanilide without a prescription, their residues may also be detected in the environment and cause reproductive harm or a restriction in cell proliferation [12,13]. Particularly, tungstate rare earth nanostructured metal oxides have drawn a lot of attention among the vast class of oxides due to their unique characteristics. Tungstate nanomaterials have grown much more appealing in recent years due to their numerous potentials uses as catalysts, solid-state lasers, photo luminescent devices, and solar electrochemical cells [14].

Due to the higher electrical conductivity of W atoms, metal tungstate nanoparticles represent an outstanding class of materials. Zinc tungsten oxide (ZnWO_4_) is the most used metal tungstate due to its superior chemical stability, increased catalytic activity, and molecular and electronic flexibility. Its band gap is 3.2 eV, due to its increased charge transfer to the catalyst, longer light reactive range, and improved carrier lifespan of the photo generated electron-hole pair. Furthermore, ZnWO_4_ nanomaterials find novel applications in sectors including luminescence, magnetic, photo electrocatalysis, and photocatalysis [15]. It has strong ultraviolet (UV) light responsiveness, adjustable band edges, optical transparency. The ease of supply, chemical stability, and sufficient strength of ZnWO_4_ has drawn a lot of interest [16]. Through the use of the proper modifications, such as hetero structure creation, doping/combining with transition metal ions, and noble metals., the band edge tanning of 4-centered nanostructures may be organized. Due to its special physical and chemical characteristics, ZnWO_4_ in particular has drawn attention. It has a wide range of possible applications, including as a scintillator material, in photoluminescence, and as photovoltaic property, humidity sensor, hydrogen sensor, and photo catalyst. Therefore, ZnWO_4_ based on binary tungstate might be a great photo catalyst for oxidizing water or filtering out hazardous contaminants from water sources. However, the quick reunion of photo-induced electron-hole pairs restricts the catalytic activity of pure ZnWO_4_ [17,18]. Through designed alterations, ZnWO_4_ nanoparticles can change their electrical environments, which can result in fascinating catalytic characteristics. In order to advance highly effective solar light-conserving photocatalysis for the removal of harmful pollutants, ZnWO_4_ has excellent physical and chemical characteristics that should be taken into consideration [19,20].

Recently, high-performing electrochemical sensors were developed using the nanomaterial carbon black (CB) as a modifier for glassy carbon electrodes (GCE). The modified GCE can be used for the adsorptive stripping voltammetry detection of CIP. Carbon black (CB) is an amorphous carbon nanomaterial made primarily of sp^2^ hybridized carbon atoms and a trace amount of sp^3^ atoms [21,22]. Its constituent particles are organized in reticulate chains or branches, and a graphite-like crystal composition may be seen there. Because of their very wide availability, high surface area-to-volume ratios, and numerous defect sites of carbon black nanoparticles (CBNPs) have attracted great interest as a potent nanomaterial [23,24].

CB is employed in many aspects of modern life and is known for being used for printer ink, rubber reinforcements, as an active ingredient in electrically conductive materials, and as paint pigments [25,26]. It has opened up new possibilities for the creation of electrochemical sensors with low cost and outstanding electroanalytical capabilities since it is less expensive than other carbon compounds such as carbon nanotubes and graphene [27]. Typically, some research teams have employed CB to create electrodes, thin films, and composite materials. Moreover, this nanomaterial may be easily changed with other materials, such as metallic nanoparticles and polymers [28,29]. CB is analogous to other carbon nanomaterials such as graphene, carbon nanotubes (both single-walled and multi-walled), and fullerene [30,31,32]. Hence, ZnWO_4_/CB has been emphasized as an excellent modifier that takes place in the electrochemical detection of the CIP [33]. Recently, GCE has been widely used due to its low residual current in aqueous media, is widely used in aqueous micellar solutions and is frequently used as an indicator. Several papers on glassy carbon electrodes have emphasized the extraordinary sensitivity of the apparent rates of redox reactions to the quality of the electrode surface, and this is the reason that we have chosen CB/ZnWO_4_/GCE for the detection of CIP [34].

Electrochemical sensors have improved the performance of conventional analytical equipment and generated low-cost substitutes by obviating the requirement for expensive reagents and delayed preparation. Since electrochemical sensors are inexpensive, portable, and simple to use, they have significant advantages over conventional analytical equipment. Electrochemical detection has advantages, such as high sensitivity, simple operation, great selectivity, short reaction time, low cost, and speedy detection. On the other hand, electrochemically active interferences in the sample, poor long-term stability, and difficult electron-transfer routes are the disadvantages of electrochemical sensors. These detection technologies also have several limitations, such as expensive electrode raw materials, environmental pollution, and lengthy electrode preparation times during the electrode material synthesis. However, electrochemical sensors, have a wide range of applications in food analysis, environmental monitoring, and clinical diagnostics. Therefore, we preferred the electrochemical method for detecting CIP by CB/ZnWO_4_/GCE [35]. The resulting nanocomposites exhibited extremely high catalytic activity and have a broad linear range, a low detection limit, good selectivity, stability, and repeatability. As a result, it is also used in the examination of practical applications using CB/ZnWO_4_/GCE towards CIP in diverse biological and environmental materials.

## 2. Experimental Section

### 2.1. Chemicals

Zinc nitrate hexahydrate (Zn(NO_3_)_2_·6H_2_O ≥ 98.0%), sodium tungstate dihydrate (Na_2_WO_4_·2H_2_O ≥ 99.0%), carbon black, sodium hydroxide (NaOH ≥ 98.0%), Ciprofloxacin and ethanol (C_2_H_5_OH) were purchased from Sigma Aldrich chemical company, Taipei, Taiwan. Here, CB nanomaterials were used as a nanocomposite with the combination of prepared ZnWO_4_. The phosphate buffer solution (PBS; 0.05 M) was prepared by Na_2_HPO_4_ and NaH_2_PO_4_ with regulations of 0.1 M H_2_SO_4_/NaOH. Deionized water was utilized to prepare all solutions and all electrochemical studies.

### 2.2. Preparation of ZnWO_4_/CB

**Scheme 1:**
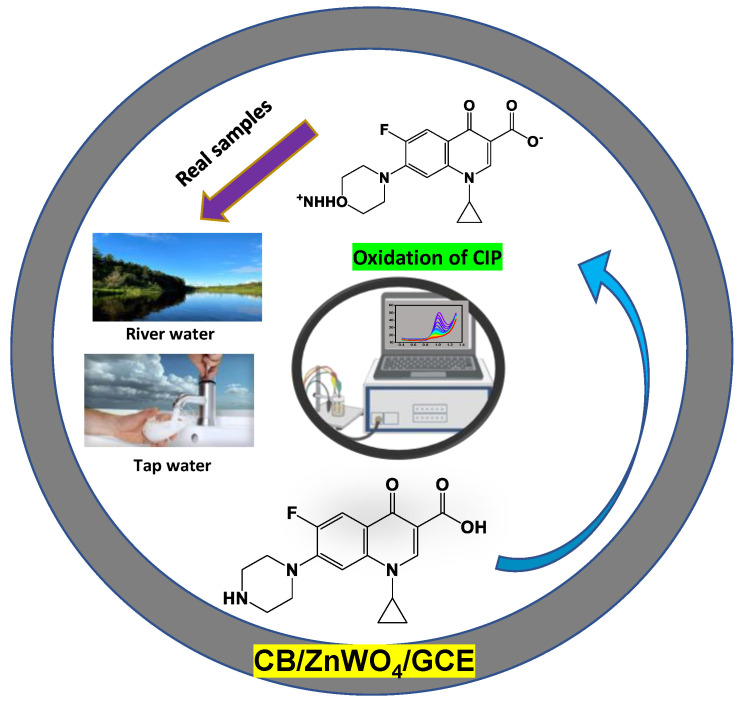
Graphical abstract of overall work of GCE/ZnWO_4_/CB electrode for CIP sensor.

The ZnWO_4_/CB composite was prepared by the simple hydrothermal method. In the common preparation process, Zinc nitrate hexahydrate (0.37 g) and sodium tungstate dihydrate (0.58 g) were dissolved with 40 mL of deionized water and the mixture was stirred continuously for 25 min. Then, 10 mL of NaOH (0.5 M) solution was mixed with the solution and it was stirred by magnetic stirring for another 20 min at room temperature. Finally, the precipitate was transferred into the Teflon-coated autoclave and kept for hydrothermal synthesis for 12 hrs with a temperature of 180 °C, after completing the reaction solution was centrifuged with C_2_H_5_OH/H_2_O. The synthesized ZnWO_4_ sample was calcinated at 400 °C for 3 h and the fine powder was collected. The prepared ZnWO_4_ was prepared with the combination of CB as ZnWO_4_/CB for the experimental procedures to obtain effective electrochemical results (Scheme 1).

### 2.3. Characterizations

The prepared samples were characterized by X-ray diffraction (XRD) which was conducted on an XPERT-PRO, PAN analytical B.V., (Netherlands) with Cu Kα radiation (k = 1.54 Å) in a range from 10° to 90° with a scan rate of 5.0° per minute. The structure, morphology, and elemental results were characterized by high-resolution transmission electron microscopy (HRTEM, JEOL JEM-2100F) and field emission scanning electron microscopy (FESEM; JEOLJSM-6500F) including energy-dispersive X-ray (EDX) studies, X-ray photoelectron spectroscopy (XPS) images were analyzed by a Thermo scientific multi-lab 2000. Electrochemical impedance spectroscopy (EIS) was recorded by the ZAHNER-Elektrik. The electrochemical methods were conducted by the CHI 1205B and CHI 900 electrochemical workstations used in the CV and DPV techniques. A glassy carbon electrode (GCE) (0.0729 cm^2^), Ag/AgCl saturated KCl, and Pt wire corresponds to the working electrode, reference electrode, and counter electrode, respectively.

### 2.4. Modification of the GCE/ZnWO_4_/CB Electrode

**Scheme 2:**
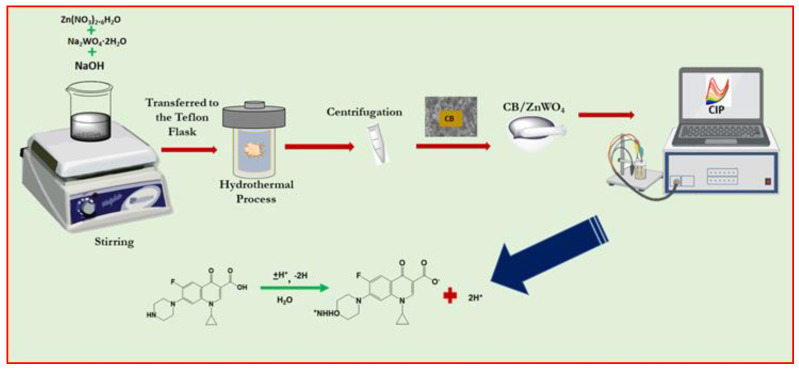
Preparation of ZnWO_4_/CB/GCE for detection of CIP.

The GCE was cleaned with water and ethanol before being polished with alumina slurries for the sensor studies. The substance was then combined with 0.5 mL of deionized water and 2.0 mg of the ZnWO_4_/CB material to create a composite while being sonicated. To fabricate ZnWO_4_/CB modified GCE, 8.0μL of ZnWO_4_/CB composite was drop cast over the precleaned GCE surface and dried for 15 min at 40 °C in an air oven. Finally, all the electrochemical experiments were performed using the constructed working electrode for the detection of CIP described in Scheme 2.

## 3. Result and Discussion

### 3.1. Characterization

**Figure 1:**
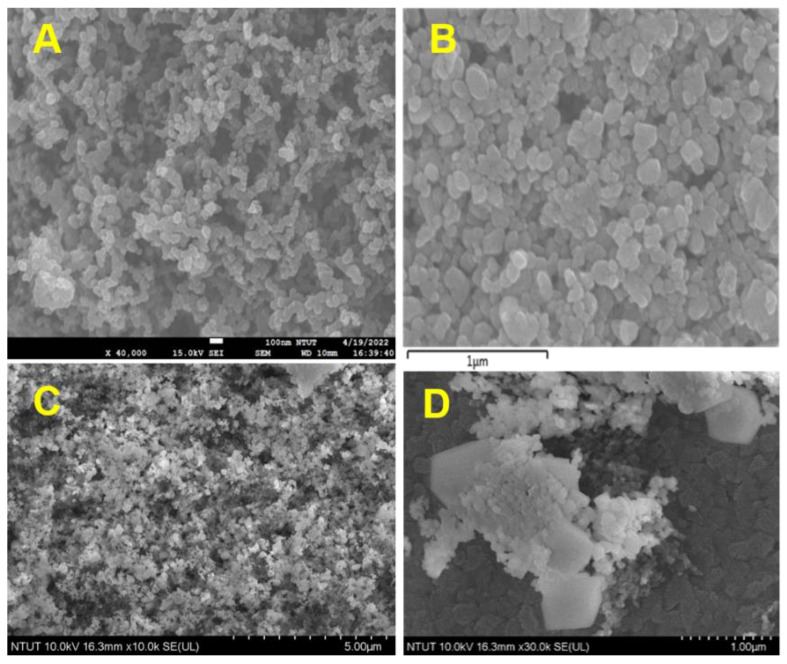
(**A**) FE-SEM image of CB; (**B**) ZnWO_4,_; and (**C**,**D**) ZnWO_4_/CB composite.

**Figure 2:**
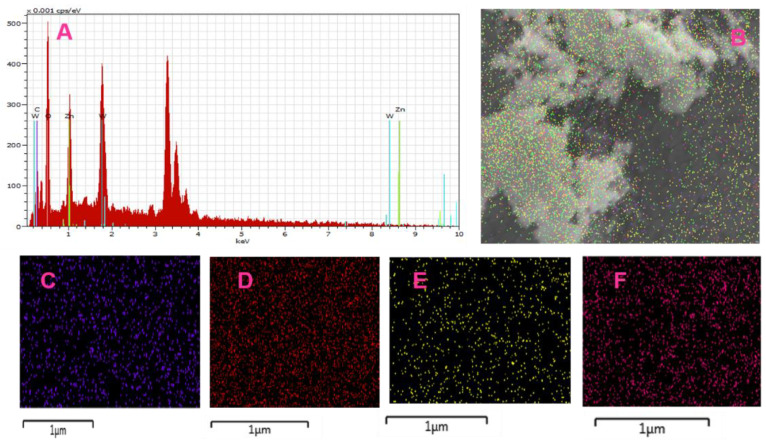
(**A**) EDAX; (**B**) elemental mapping of ZnWO_4_/CB composite; (**C**) Zn; (**D**) W; (**E**) O; and (**F**) C.

**Figure 3:**
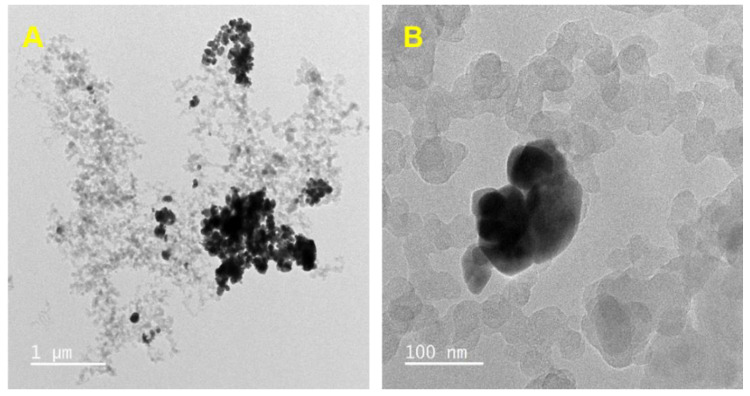
(**A**,**B**) TEM image of ZnWO_4_/CB composite.

FE-SEM, EDAX, and TEM analysis were used to examine the surface morphology of pure CB, ZnWO_4,_ and ZnWO_4_/CB composites. Figure 1A shows the SEM image of CB has elemental carbon organized into tiny particles to create the CB primary particle, which has an amorphous and quasi-graphitic structure. However, the characteristics of CB are nanostructured aggregates exhibiting semispherical groups. Additionally, these aggregate groupings have a defined long dimension. The range of CB particle sizes is 3.0 to 100 nm. The ZnWO_4_ nanoparticles were uniformly sized at 100 nm, and show a monoclinic crystal system in Figure 1B. Figure 1C,D exhibits the FE-SEM pictures of the ZnWO_4_/CB composite, the ZnWO_4_ nanoparticles were adorned on CB to form the ZnWO_4_/CB composite. In addition, energy dispersive X-ray spectra and elemental mapping analysis studies were used to study the chemical composition and distribution of the ZnWO4/CB composite (Figure 2A). Figure 2B shows the EDS spectrum found in Zinc (Zn), tungsten (W), carbon (C), and oxygen (O) components, without any other contaminants. Additionally, Figure 2C–F demonstrates the elemental mapping results which show that the Zn, W, C, and O in the scanning area were distributed uniformly throughout the ZnWO4/CB composite. Figure 1C shows that the elemental mapping exhibiting the ZnWO_4_ particles was uniformly distributed on the surface of CB. The EDAX studies depict the element weight with the percentages of Zn as 21.6%, Was 62.8%, O as 15.6%, and C as 100%, respectively [36]. Figure 3A,B shows the prepared nanocomposites further studied with TEM analysis, which shows that the ZnWO_4_ particles coated on the CB surface and a well-formed ZnWO_4_/CB composite.

**Figure 4:**
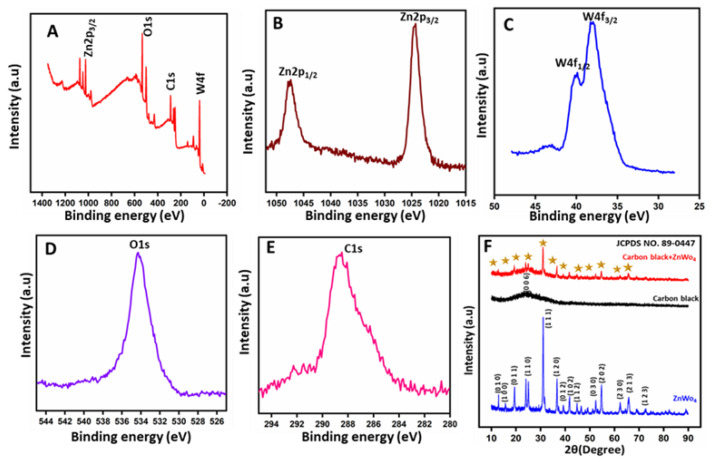
(**A**) XPS survey spectrum of ZnWO_4_/CB; (**B**) Zn 2p; (**C**) W 4f; (**D**) O 1s; (**E**) C 1s; and (**F**) XRD pattern of CB, ZnWO_4_, ZnWO_4_/CB composite.

Figure 4A shows the XPS studies done on the ZnWO_4_/CB composite, which indicate the survey scan of elements such as Zn 2p, W 4f, O 1s, and C 1s. The absorption of air atmosphere in the prepared sample shows the C element peak. The spectra of Zn 2p^1/2^ and Zn 2p^3/2^ are displayed in Figure 4B with binding energies of 1046.5 eV and 1045.6 eV. Figure 4C shows that W4f^1/2^ and W4f^3/2^ are ascribed to the corresponding peaks at 40.5 eV and 46.6 eV, respectively. Figure 4D shows that the O1s peaks at 534 eV are attributed to the metal–oxygen bond and the hydroxyl group that was absorbed from the moisture. Figure 4E shows that the binding energies C 1s peaks at 288 eV [36]. XPS studies confirmed the successful formation of the ZnWO_4_/CB composite.

Figure 4F shows the XRD pattern of the CB, ZnWO_4_, and ZnWO_4_/CB composite. The cubic point of the space group of ZnWO_4_/CB contains diffraction peaks at an angle of 2θ = 15.47°, 18.89°, 23.81°, 24.51°, 30.70°, 36.76°, 39.94°, 37.80°, 41.56°, 44.59°, 47.64°, 52.74°, 54.24°, 62.73°, 65.47°, 69.32°, 72.45° and 26.70° that corresponds to the (010), (100), (011), (110), (111), (120), (012), (102), (112), (030), (122), (020), (230), (023), (23) and (006) crystal planes, respectively (JCPDS-01-089-0447). These findings demonstrated that ZnWO_4_ and CB diffraction peaks successfully appeared in the ZnWO_4_/CB composite composition without impurities, which confirms the formation of the ZnWO_4_/CB composite [37].

### 3.2. Electrochemical Performance of Different Modified Electrodes

**Figure 5:**
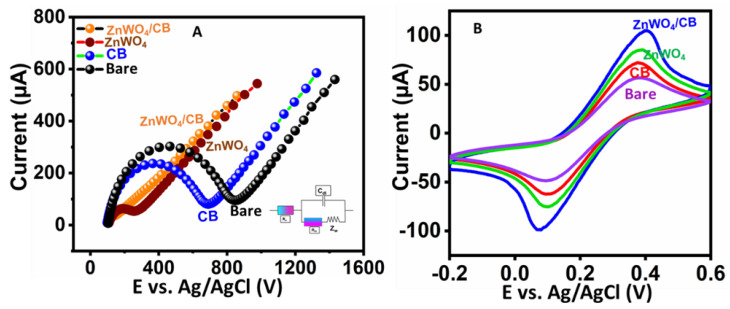
(**A**) EIS; and (**B**) CV studies of bare GCE, CB/GCE, ZnWO_4_/GCE, and GCE/ZnWO_4_/CB were performed by 0.1 M KCl with 5.0 mM [Fe(CN)_6_]^3−/4−^.

The interfacial resistance of the GCE, CB/GCE, ZnWO_4_/GCE, and ZnWO_4_/CB/GCE was investigated using electrochemical impedance spectra (EIS) utilizing a solution of 0.1 M KCl and 5.0 mM [Fe(CN)_6_]^3−/4−^ at the detected potential of 10 mV and a frequency range of 0.1 Hz to 100 kHz. Figure 5A shows the EIS plot in two sections that are consistent with each other. The high-frequency semicircle region, which is associated with charge transfer resistance (*R*_ct_), and the low-frequency linear zone are related to diffusion kinetics. The bare GCE, CB/GCE, ZnWO_4_/GCE, and ZnWO_4_/CB/GCE have identified the *R*_ct_ values of 745.7 Ω, 586.9 Ω,149.21 Ω, and 44.43 Ω, respectively.

Figure 5B shows the CV profiles of different modified electrodes using 0.1 M KCl and 5.0 mM [Fe (CN)_6_]^−3/−4^ at 50 mVs. All the modified electrodes had redox peaks. In comparison to the other electrodes, the bare GCE electrode exhibits the lowest peak-to-peak separation (ΔE_p_ = 0.34 V) and the reversible current response (*I*_pa_ = 40.38 μA and I_pc_ = −40.32 μA) for the bare electrode, as well as the estimated values of ZnWO_4_/GCE (ΔEp = 0.45 V) and GCE/ZnWO_4_/CB, has (*I*_pa_ = 103.38 μA and *I*_pc_ = −103.32 μA). Hence, the lower E_p_ and higher redox peak currents indicate that the GCE/ZnWO_4_/CB has a greater electroactive surface area and transfers electrons more quickly than the other bare GCE, CB/GCE, and ZnWO_4_/GCE electrodes. Additionally, the GCE/ZnWO_4_/CB composite display a significantly increased redox peak current as compared to bare GCE, GCE/CB, and GCE/ZnWO_4_. As shown by these results, the enhanced GCE/ZnWO_4_/CB electrode exhibits strong conductive properties that promote higher charge transfer efficiency.

### 3.3. Electrochemical Oxidation of Various Modified Electrodes and Different pH

**Figure 6:**
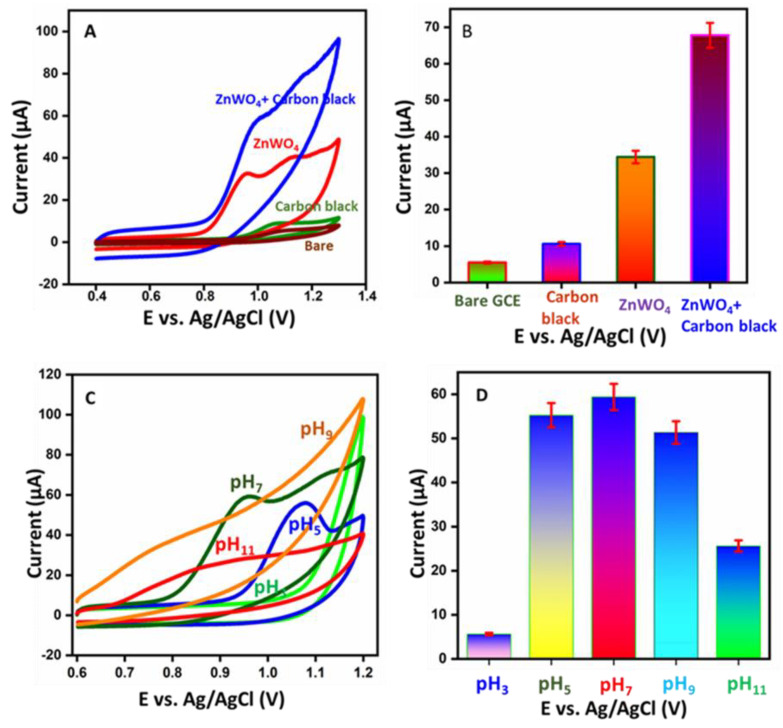
(**A**) CV studies of the bare GCE, GCE/CB, GCE/ZnWO_4_, and GCE/ZnWO_4_/CB in 100 μM CIP; (**B**) the bar graph for various modified electrodes, (**C**) CV measurements of GCE/ZnWO_4_/CB at various pH values (0.05 M PBS) at nitrogen immersed 100 µM CIP; and (**D**) the bar diagram for different pH graph of GCE/ZnWO_4_/CB for electrochemical detection of CIP.

**Scheme 3:**
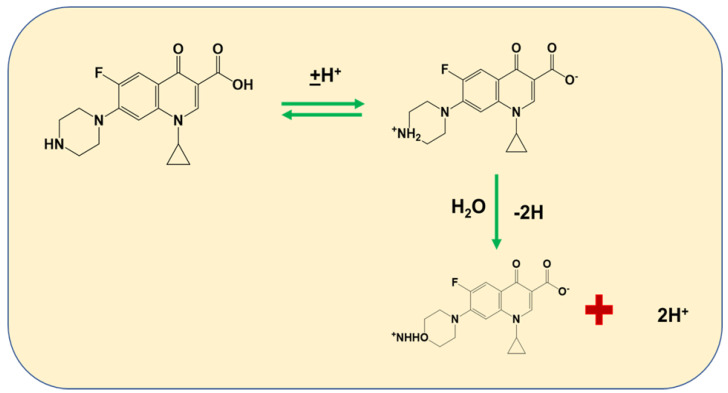
Electrochemical oxidation of CIP by GCE/ZnWO_4_/CB electrode.

Figure 6A shows the electrochemical oxidation of CIP was studied by CV at 0.05 V s^−1^ with pH 7.0 on applied potentials starting from 0.4 V to 1.4 V with the bare GCE, GCE/CB, GCE/ZnWO_4_, and GCE/ZnWO_4_/CB. The current response in the 0.05 M PBS electrolyte solution with and without the addition of 100 µM of CIP and also represented by the bar diagram is shown in Figure 6A,B. On this electrode, a substantial oxidation peak current was determined. As can be seen, there is no oxidation peak current seen for the GCE/ZnWO_4_/CB when CIP is not present. The lowest oxidation peak current on the addition of CIP was observed at *I*_pa_ = 5.2 μA with an oxidation peak potential of (ΔE_p_) 1.02 V (bare GCE). After CB was coated on the GCE surface, oxidation peaks current of *I*_pa_ = 8.51 μA along a peak potential of *E*_pa_ = 1.05 V was obtained. Additionally, the ZnWO_4_/ GCE electrode compared to the bare GCE and CB/GCE exhibits a moderately improved oxidation peak current and a positively transferred oxidation potential of (*E*_pa_ = 1.48 V) and has *I*_pa_ = 30.53 μA with a peak potential of *E*_pa_ = 0.85 V. As a result, GCE/ ZnWO_4_/CB has exceptional electrical conductivity and excellent catalytic function for detecting CIP. When compared to the bare GCE, CB/GCE, and ZnWO_4_/GCE, the fabricated GCE/ZnWO_4_/ CB showed the highest oxidation peak current response at *I*_pa_ = 60.9 μA, and the oxidation peak potential shifted to the more positive side (*E*_pa_ = 0.93 V). In contrast to the other electrodes, the modified GCE/ZnWO_4_/CB exhibits exceptional electrochemical activity and a rapid electron shifting process during CIP detection (Scheme 3).

By the different pH ranging from 3.0 to 11.0, the impact of pH on the electrocatalytic behavior of CIP in 0.05 M PBS at 0.05 Vs^−1^ was determined by GCE/ZnWO_4_/CB electrode and shown in Figure 6C,D. An anodic peak current appeared from pH 3.0 to 11.0 upon the addition of 100 µM of CIP. As can be seen, as the pH rises from 3.0 to 7.0, the amplification of CIP steadily increases because there are more protons freely circulating. The electrolyte solution of GCE/ZnWO_4_/CB has *I*_pa_ = 60.13 μA with a peak potential of *E*_pa_ = 0.83 V. Hence, the oxidation peaks current increases with pH above 7.0, probably as a result of the hydrogen ions interfering with CIP detection. Finally, the very quick decrease in peak currents in the pH range of 7.0–11.0. The pH of the CIP was then influenced, and the peak current shift was obtained. Thus, we have chosen pH 7.0 for further electrochemical studies. The CIP oxidized to the formation of cationic radical and the single electron transfer kinetics on the GCE/ZnWO_4_/CB electrode has occurred.

### 3.4. Effect of Different Concentrations and the Scan Rates

**Figure 7:**
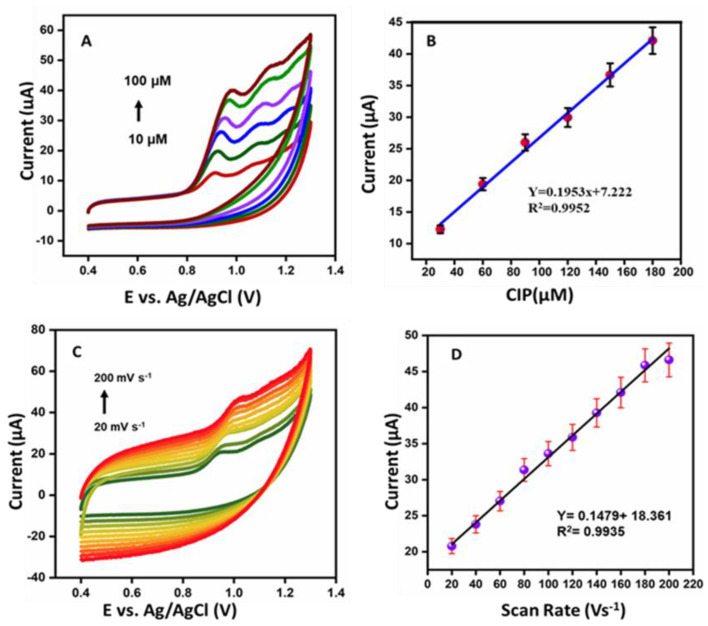
(**A**) CV curve of GCE/ZnWO_4_/CB with various additions of CIP (0–100 μM) at 50 mVs^−1^; (**B**) linear plots of current (*I*_pa_) vs. other concentrations of CIP; (**C**) CV curves of the altered electrode GCE/ZnWO_4_/CB towards 100 µM CIP at various sweep rates (20–200 mV s^–1^); and (**D**) The graph between the square root of scan rate (v^1/2^) and anodic peak current was done with N_2_ immersed pH 7.0 (0.05 M PBS).

The CV response at a scan rate of 50 mV s^−1^ for CIP concentrations ranging from 0–100 µM at GCE/ZnWO_4_/CB in pH 7.0 (50 mM PBS). From 0 to 100 µM, the oxidation peaks current (*I*_pa_) gradually increased concerning the various concentrations in Figure 7A and the mechanism of oxidation of CIP is shown in Scheme 3. The calibration curve between the oxidation peak current and various concentrations is shown in Figure 7B, *I*_pa_ (µA) = 0.1953X [µM] + 7.222, (R^2^ = 0.9952) is the corresponding regression equation and correlation coefficient which is represented in Figure 7B. However, the findings show that the GCE/ZnWO_4_/CB has excellent electrocatalytic activity towards CIP detection as well as it has quick electron transfer. The CV was carried out with GCE/ZnWO_4_/CB in various scan rate ranges of 20–200 mVs^−1^ and reached pH 7.0, as shown in Figure 7C, to test the various scan rates of CIP. As the sweeping scan rate is increased, the anodic peak current of CIP progressively rises. The relation between anodic peak current and scan rate is presented in Figure 7C. Figure 7D shows the linear regression equation is *I*_pa_(µA) = 0.1479 × (mVs^−1^) + 18.361 and the coefficient of (*R*^2^ = 0.9935). This finding implies that diffusion controls the electrocatalytic oxidation of CIP detected on the GCE/ZnWO_4_/CB surface [37].

### 3.5. Differential Pulse Voltammetry (DPV), Interference Studies, and Repeatability of CIP

**Figure 8:**
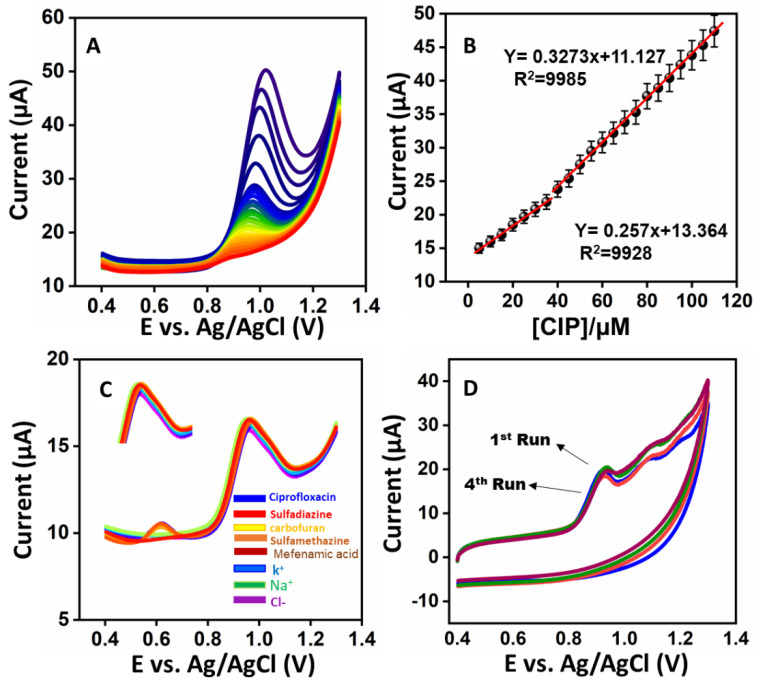
(**A**) DPV measurements of GCE/ZnWO_4_/CB with various addition of CIP range from 0.02 to 120 µM with N_2_ immersed in 0.05 M PBS (pH 7); (**B**) linear graph along oxidation peak current and different concentration of CIP); (**C**) interference studies of various interrupting compounds; and (**D**) repeatability study with N_2_ immersed in 0.05 M PBS (pH 7).

DPV has higher sensitivity, greater resolution, and better selectivity when compared to other voltammetry methods. In order to ascertain the reduced concentration of CIP on GCE/ZnWO_4_/CB in 0.05 M PBS (pH 7) containment, we chose the DPV approach, which is shown in Figure 8A. As the CIP concentration is increased from 0.01 µM to 120 µM, the oxidation peaks current rises linearly, as seen in the linear plot of the maximum current during oxidation vs. various CIP concentrations. We obtained three linear ranges. The linear calibration equation observed for the first linear range from 0.01 µM to 120 µM has *I*_pa_ (µA) = 0.3273 C (µM) + 11.127 (R^2^ = 9985) and is shown in Figure 8B. The analytical parameters from the first linear range were computed, with the calculated limit of detection being 0.02 µM and sensitivity being 1.71 µA µM^−1^ cm^−2^. The following equation was used for the calculation [38]

𝐿𝑂𝐷 = 3σ/S(1)

Here S denotes the linear plot’s slope value and denotes the relative standard deviation (RSD). The acquired analytical properties were compared to those of previously reported sensors and included a wide linear range, good sensitivity, and a lower detection limit of CIP on GCE/ZnWO_4_/CB, which was calculated by Equation (1) [39]. GCE/ZnWO_4_/CB has received a lot of attention lately, due to their distinct optical, physical, and electrochemical applications as well as their prospective advantages in terms of higher surface area, improved chemical stability, equal particle size distribution, and high porosity. For electrochemically detecting CIP, we have created a ZnWO_4_ nanomaterial that exhibits a broad range of potential and has an outstanding catalytic activity. However, the detection response of the CIP at the CB/ZnWO4/GCE is lower than that of the other electrochemical sensor mentioned in Table 1 such as, TiO_2_/AuNPs/Nafion/CMK-3 electrode (LOD = 0.108 μM) [7], AuNP/CHI/SPCE electrode (LOD = 0.001 μM) [8], CNT-V_2_O_5_CS/SPCE electrode (LOD = 3.0 μM) [10], PBE electrode (LOD 4.96 μM) [40], TiO_2_/PVA-GCE electrode (LOD = 0.04 μM) [41], GR-ZnO/GCE electrode (LOD = 0.4 μM) [42], GO/ZnO/GCE electrode (LOD = 0.01 μM) [43], and OLA-Fe_3_O_4_/MWCNTs/GCE electrode (LOD = 0.06 M) [44]. Moreover, the CB/ZnWO_4_/GCE electrode exhibited CIP detection at a low detection limit and long linear range than another electrode due to the high activity and active surface area of the CB/ZnWO_4_/GCE. Therefore, this result demonstrates that the CB/ZnWO_4_/GCE can be used as an excellent electrode for the sensing of CIP.

DPV was conducted to determine the impact of varying possible anti-interference investigations in 0.05 PBS (pH 7) with 20 µM CIP at GCE/ZnWO_4_/CB. As can be shown in Figure 8C, CIP was selectively detected at the same time, with the oxidation peak potential being 1.26 V. The initial oxidation peak current and peak potential of the original CIP were unaffected by other interfering compounds. Furthermore, CIP with the presence of various interferents such as sulfadiazine, carbofuran, sulfamethazine, mefenamic acid, K^+^, Cl^−^, and Na^+^ were studied and shown in Figure 8C. The experiments were conducted in N_2_-immersed conditions with a pH of 7.0 (0.05 M PBS) and 0.05 V s^−1^. CIP’s independence from outside meddling has thus been demonstrated in Figure 8C. Additionally, the CV examination of the manufactured electrodes in the assessment of repeatability for the CIP sensor was successful and are shown in Figure 8D. With a critical current difference between the oxidation peak current of 100 µM CIP in 0.05 M PBS (pH 7), it can be concluded that the GCE/ZnWO_4_/CB had better sensor repeatability.

### 3.6. Reproducibility, Stability, and Real Sample Analysis

**Figure 9:**
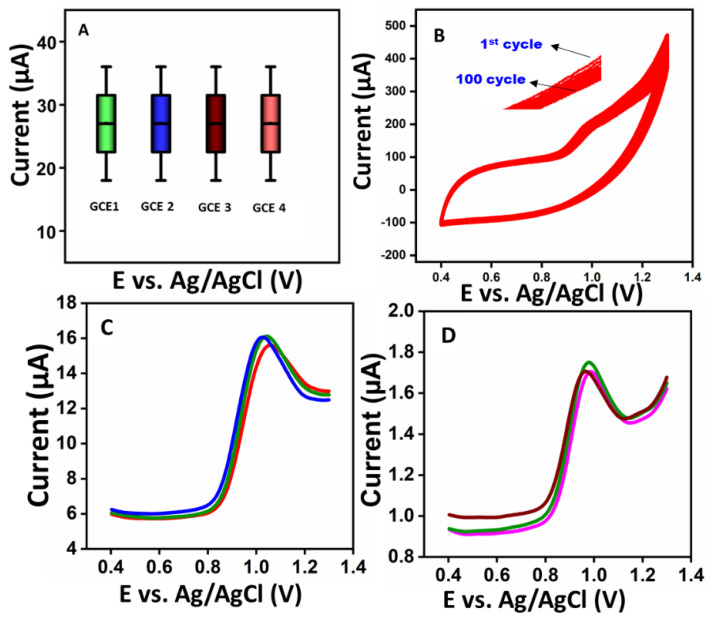
(**A**) Reproducibility studies of various CB/ZnWO4/GCE electrodes; (**B**) stability storage studies of GCE/ZnWO_4_/CB with CIP. DPV response of real samples such as river water; (**C**) and tap water; and (**D**) in 0.05 M PBS (pH 7) with various amounts of CIP.

The reproducibility of GCE/ZnWO_4_/CB was tested in four separate tests using a different electrode in 100 µM CIP containing 0.05 M PBS (pH 7) and the results are shown in Figure 9A. We suggest that the manufactured sensor GCE/ZnWO_4_/CB has an outstanding level of reproducibility. Furthermore, the stability of storage was studied by the CV in N_2_ immersed in 0.05 M PBS (pH 7) containing 100 µM CIP at the extensive scan rate of 0.05 Vs^−1^ by the optimal circumstances shown in Figure 9B. The altered electrode current response shows very good stability as well as the electrocatalytic oxidizing property of CIP.

The presence of CIP in real samples taken from various rivers and tap water was detected by the GCE/ZnWO_4_/CB electrode (Figure 9C,D). As a result, real samples of the undefined concentration were spiked with the known concentration of CIP mentioned in Table 2. The recovery values exhibit the relative standard deviation (RSD) approach. These results represent the experimental applications of the GCE/ZnWO_4_/CB altered electrode for the determination of CIP in real samples of water with excellent recoveries. The recoveries values of river water and tap water samples were obtained as 98.5% and 99.6%, with average RSD values of 4.2% and 3.6%, respectively. The results of real sample analysis demonstrated the outstanding capability of GCE/ZnWO_4_/CB electrodes for the detection of the CIP. Therefore, the values found are very reasonable for use in practical applications.

## 4. Conclusions

In this study, we effectively prepared a reliable, simple, and affordable GCE/ZnWO_4_/CB electrode for the electrochemical detection of CIP. The crystalline structure and shape of the prepared materials were confirmed by the analytical spectroscopic characterizations of XRD, FTIR, FESEM, and EDX-elemental mapping. The GCE/ZnWO_4_/ CB electrode shows high sensitivity (1.71 μA μM^−1^ cm^−2^) with a lower detection limit of 0.02 µM, and the electrochemical performance of the modified GCE showed a long linear range from 0.02 to 120 µM, with effective electrocatalytic responsiveness of CIP detection. Furthermore, the suggested electrode has higher selectivity, stability, repeatability, and reproducibility. The GCE/ZnWO_4_/CB exhibited an excellent result for the electrochemical measurement of CIP in real water samples. Therefore, the GCE/ZnWO_4_/CB electrode is also a promising option for the electrochemical detection of hazardous chemicals in biological and environmental samples.

## Figures and Tables

**Table 1:** Comparison by modified GCE/ZnWO_4_/CB electrode with different sensors in CIP detection.

Different Electrodes	Linear Range (µM)	LOD (µM)	References
^a^ TiO_2_/^b^ AuNPs/Nafion/^c^ CMK-3		0.108	[7]
^d^ AuNP/^e^ CHI/^f^ SPCE	-	0.001	[8]
^g^ CNT-^h^ V_2_O_5_CS/^i^ SPCE	-	3.0	[10]
^j^ PBE	-	4.96	[40]
TiO_2_/^k^ PVA-GCE	10–120	0.04	[41]
^l^ GR-^m^ ZnO/GCE	1–180	0.4	[42]
^n^ GO/ZnO/GCE	0.2–7.2	0.01	[43]
^o^ OLA-^p^ Fe_3_O4/^q^ MWCNTs/GCE	0.01–8.9	0.06	[44]
^r^ SDS·^s^ Gr/^t^ CPE	0.3–100	0.029	[45]
^u^ GCE/^v^ ZnWO_4_/^w^ CB	0.02–120	0.02	This work

^a^ Titanium dioxide, ^b^ Gold Nanoparticles, ^c^ mesoporous carbon, ^d^ Gold nanoparticles, ^e^ Chitosan, ^f^ Screen printed electrodes, ^g^ carbon nanotubes, ^h^ vanadium pentoxide-chitosan, ^i^ screen-printed carbon electrodes, ^j^ Paper based electrodes, ^k^ polyvinyl alcohol, ^l^ graphene, ^m^ zinc oxide, ^n^ graphene oxide, ^o^ Oleylamine, ^p^ Iron Oxide, ^q^ Multi-walled carbon nanotubes, ^r^ sodium dodecyl sulfate, ^s^ graphene, ^t^ Carbon paste electrodes, ^u^ Glassy carbon electrode, ^v^ Zinc tungsten oxide, ^w^ carbon black.

**Table 2:** Outcomes for the detection of CIP in various real samples.

Real Samples	Added	Found	Recovery (%)	RSD (%)
River water	10	9	98.5	
	20	19	98.1	4.20%
	30	29	97.6	
Tap water	10	9.5	99.2	
	20	19	98.5	3.60%
	30	29.5	98.4	

## Data Availability

Not applicable.
